# Methane oxidation in anoxic lake water stimulated by nitrate and sulfate addition

**DOI:** 10.1111/1462-2920.14886

**Published:** 2020-01-01

**Authors:** Sigrid van Grinsven, Jaap S. Sinninghe Damsté, Alejandro Abdala Asbun, Julia C. Engelmann, John Harrison, Laura Villanueva

**Affiliations:** ^1^ Department of Marine Microbiology and Biogeochemistry NIOZ Royal Netherlands Institute for Sea Research, and Utrecht University Den Burg The Netherlands; ^2^ Department of Earth Sciences, Faculty of Geosciences Utrecht University Utrecht The Netherlands; ^3^ Washington State University Vancouver, School of the Environment Vancouver WA 98686 USA

## Abstract

Methanotrophic bacteria play a key role in limiting methane emissions from lakes. It is generally assumed that methanotrophic bacteria are mostly active at the oxic‐anoxic transition zone in stratified lakes, where they use oxygen to oxidize methane. Here, we describe a methanotroph of the genera *Methylobacter* that is performing high‐rate (up to 72 μM day^−1^) methane oxidation in the anoxic hypolimnion of the temperate Lacamas Lake (Washington, USA), stimulated by both nitrate and sulfate addition. Oxic and anoxic incubations both showed active methane oxidation by a *Methylobacter* species, with anoxic rates being threefold higher. In anoxic incubations, *Methylobacter* cell numbers increased almost two orders of magnitude within 3 days, suggesting that this specific *Methylobacter* species is a facultative anaerobe with a rapid response capability. Genomic analysis revealed adaptations to oxygen‐limitation as well as pathways for mixed‐acid fermentation and H_2_ production. The denitrification pathway was incomplete, lacking the genes *narG/napA* and *nosZ*, allowing only for methane oxidation coupled to nitrite‐reduction. Our data suggest that *Methylobacter* can be an important driver of the conversion of methane in oxygen‐limited lake systems and potentially use alternative electron acceptors or fermentation to remain active under oxygen‐depleted conditions.

## Introduction

The concentration of atmospheric methane, a potent greenhouse gas, has increased strongly since the pre‐industrial era. Knittel and Boetius (2009) estimated that 10–20% of all reactive organic matter buried in sediments is converted to methane. For the oceans, this is estimated to be 85–300 Tg of methane per year. Freshwater lakes and reservoirs have been a long‐overlooked source of methane, but recent research has shown their importance for the global methane budget (Bastviken *et al*., 2011; Deemer *et al*., 2016). Methane production in shallow lakes may further increase as a result of increasing air and water temperatures due to global warming (Marotta *et al*., 2014).

Methane emissions are strongly limited by both aerobic and anaerobic methane oxidation. Marine methane oxidation is generally performed by a consortium of anaerobic methane‐oxidizing archaea (ANME) and sulfate‐reducing bacteria, which use the widely abundant sulfate as a terminal electron acceptor (Reeburgh, 2007). Recently, iron and manganese oxides have also been suggested to function as potential electron acceptors for methane oxidation in brackish sediments (Egger *et al*., 2014; Ettwig *et al*., 2016). In freshwater systems and wetland soils, microaerobic methane oxidation at oxic‐anoxic interfaces is a major pathway for methane removal both in the sediment and in the water column (Rudd *et al*., 1976). Freshwater anaerobic oxidation of methane (AOM) with sulfate has been suggested to take place in freshwater sediments (Schubert *et al*., 2011). Both nitrate and nitrite were used as electron acceptors for AOM in methanotrophic cultures originating from freshwater sediments or water (Raghoebarsing *et al*., 2006; Ettwig *et al*., 2010; Deutzmann and Schink, 2011; Kits *et al*., 2015b; Oswald *et al*., 2017). Iron and manganese have been shown to enhance lacustrine AOM in a Swiss lake; the responsible process could, however, not be determined (Oswald *et al*., 2016). Dissolved organic matter and humic substances can also function as electron acceptors and may be relevant for methane oxidation in eutrophic systems (Valenzuela *et al*., 2019). The humic acid analogues quinone and anthraquinone‐2,7‐disulphonate have been implicated in anaerobic methane oxidation, although a direct coupling between methane oxidation and reduction of organic material has not yet been demonstrated (Reed *et al*., 2017).

Microaerophilic methane oxidation is performed by type I (Gammaproteobacteria) or type II (Alphaproteobacteria) methane oxidizing bacteria, commonly called methanotrophs. Recently, specific methanothrophs of the genus *Methylomonas* were discovered to be facultative anaerobes, capable of methane oxidation with nitrate as the terminal electron acceptor (Kits *et al*., 2015b). Earlier, *Ca*. Methylomirabilis oxyfera, a bacterium of the NC10 division, was found to perform methane oxidation in anoxic environments by using nitrite as an electron acceptor for methane oxidation, via internal oxygen production (Ettwig *et al*., 2010). Recently, Oswald and colleagues (2017) discovered that *Crenothrix polyspora* possesses the key methane oxidizing enzyme methane monooxygenase, and that it may be an important methane oxidizer in stratified lakes. They showed *C*. *polyspora* can grow under both oxygen‐rich and oxygen‐depleted conditions, and that its genome encodes pathways for respiration of both oxygen and nitrate, suggesting it is a facultative anaerobic methane oxidizer that can couple methane oxidation to nitrate reduction. Although there is a growing recognition that methane oxidation is carried out by a diverse array of organisms utilizing a combination of aerobic and anaerobic metabolic pathways, our knowledge of these organisms, their metabolic strategies and their ecosystem effects remain poorly understood.

This study is aimed at expanding our knowledge of the potential terminal electron acceptors and the diversity and role of methanotrophs in the anoxic water column of stratified, eutrophic lakes. The anoxic water column can become a methane reservoir during stratified periods, with a potential for anaerobic methane oxidation, potentially supported by nitrate as an electron acceptor for methanotrophs. Our study site is Lacamas Lake, a seasonally stratified reservoir in Washington State, USA with methane concentrations up to 270 μM in the anoxic hypolimnion during summer. We addressed the following research questions: (i) to what extent can alternative terminal electron acceptors (i.e., not O_2_) stimulate methane oxidation in the anoxic water column, (ii) what are the responsible organisms and (iii) how do they achieve methane oxidation and biomass production even in conditions that appear unfavourable. To this end, we explored the effect of enhanced nitrate and sulfate concentrations and oxic‐anoxic conditions on methane oxidation rates in 24 and 72 h incubation experiments. The methanotrophic community was analysed by both 16S rRNA and particulate methane monoxygenase subunit A (*pmo*A) coding gene amplicon sequencing and quantitative polymerase chain reaction (qPCR). The most abundant methanotroph, a novel *Methylobacter* species, was further investigated using a metagenomic sequencing approach.

## Results

### *Physicochemical characteristics*

**Figure 1:**
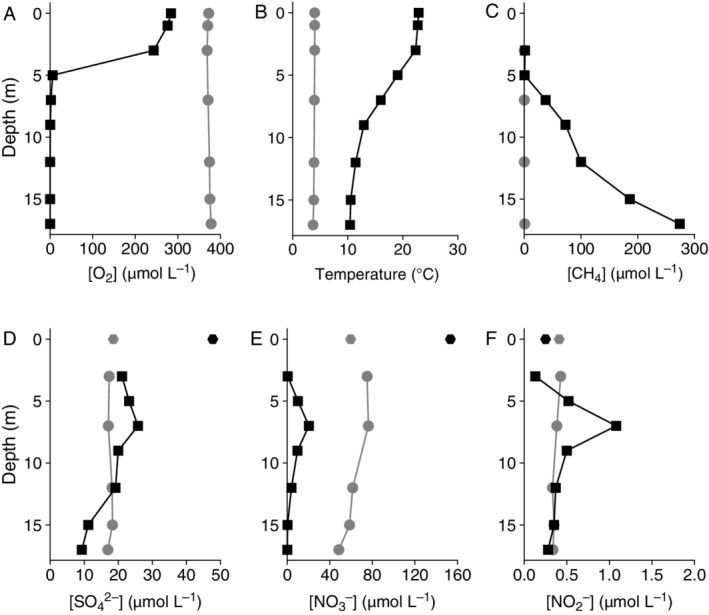
Physicochemical conditions of Lacamas Lake in August 2016 (black squares) and February 2017 (grey circles). A. Dissolved oxygen concentration. B. Temperature (°C). C. Dissolved methane concentration. D. Sulfate concentration. E. Nitrate concentration. F. Nitrite concentration. The data points at 0 m in (D), (E) and (F) represent the concentrations of sulfate, nitrate and nitrite, respectively, in the inlet stream in summer and winter, sampled 1.5 km upstream of the lake.

During our late August sampling, the lake was stratified, with an anoxic, methane‐rich (38–270 μM CH_4_; Fig. 1) hypolimnion. The oxycline was located at 3–5 m. Nitrate concentrations were 10–20 μM at 5–9 m, but < 0.5 μM in the shallow (3 m) and deep (15–17 m) waters, despite the high concentration in the inflowing stream (153 μM; Fig. 1). Sulfate concentrations were around 20 μM in the shallow water and decreased gradually to 9 μM at 17 m, lower than that of the inflowing stream (48 μM; Fig. 1). During winter sampling in February, the water column was homogeneously oxygenated and the methane concentration was low (0.2–0.8 μM; Fig. 1). Water column nitrate concentrations were 4–300 times higher than in summer (76 μM in surface waters, decreasing to 49 μM above the sediment), while the nitrate concentration in the inlet water had decreased to 60 μM. Sulfate concentrations of the winter water column were 17–18 μM throughout the water column (Fig. 1). Nitrite concentrations were low year‐round (0.1–1 μM; Fig. 1).

### *Methane oxidation rates*

**Figure 2:**
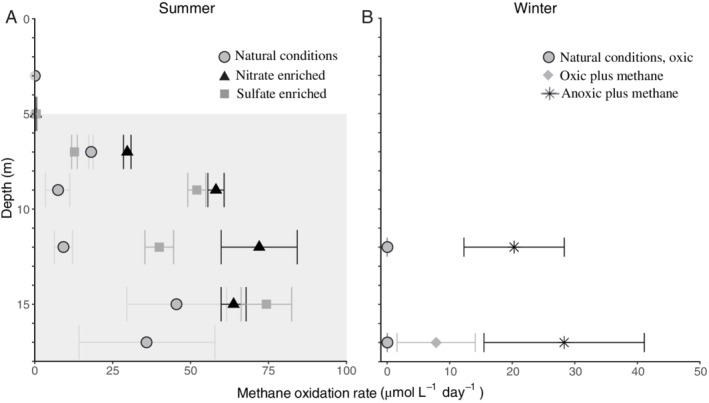
Methane oxidation rates as determined from incubation experiments in (A) summer electron acceptor experiments and (B) winter methane addition experiments. In (A), the observed methane oxidation rates in natural (unamended) summer conditions (light grey circles), nitrate enriched conditions (black triangles) and sulfate enriched conditions (dark grey squares) are shown. The grey shading indicates the anoxic zone. At 3 and 17 m depth, no enrichment experiments were performed. At 5 m depth, all methane oxidation rates were < 0.5 μM day^−1^. In (B), natural (unamended) oxic conditions (dark grey inverted triangles), oxic conditions with methane added (grey diamonds) and anoxic conditions with methane added (black stars) are shown for samples from 12 and 17 m depth. A negative rate was observed in the oxic + methane experiment at 12 m depth (results not shown). Note the different scale on both x‐axes. Details are given in Table S1.

During summer stratification, net methane oxidation was detected throughout the hypolimnion (7–17 m) with rates ranging from 7.3 to 46 μM day^−1^, peaking at 7 and 15 m (Fig. 2A, Table S1). Incubation experiments revealed that at 7 m, > 60% of the methane was oxidized within 24 h, whilst at 9–15 m this was only 6–16% (Table S1). No methane oxidation was detected in the oxic zone (3–5 m depth) of the summer water column, despite the natural presence of dissolved methane (2.2–2.7 μM; Table S1). Additions of nitrate (10x natural concentration, 74–146 μM) and sulfate (100x natural concentration, up to 2.2 mM) increased the methane oxidation rate at all measured depths (5–15 m) except for 7 m, where methane was limiting. Nitrate and sulfate stimulated rates were up to eight times higher than control rates, up to 72 μM day^−1^ at 12 m with nitrate added, and up to 74 μM day^−1^ at 15 m with sulfate added (Fig. 2A). With the addition of humic substances and oxygen to the anoxic waters at 12 m, methane oxidation completely diminished (Fig. S1).

In winter, methane concentrations in the water column were < 1 μM and no methane oxidation was detected (Figs 1 and 2B). To test the potential for methane oxidation, methane concentrations were increased to 150–260 μM, corresponding to concentrations in the hypolimnion in summer (Table S1), and part of the incubations were performed under anoxic conditions (see *Experimental Procedures* for details). The potential methane oxidation rate under anoxic conditions was 37 μM day^−1^, three times higher than in oxic methane‐amended conditions at 17 m depth (Fig. 2B). At 12 m, methane was oxidized at a rate of 20 μM day^−1^ under anoxic conditions, whilst the methane concentration increased over time in oxic conditions.

### *Identity and abundance of methanotrophs in the water column*

**Figure 3:**
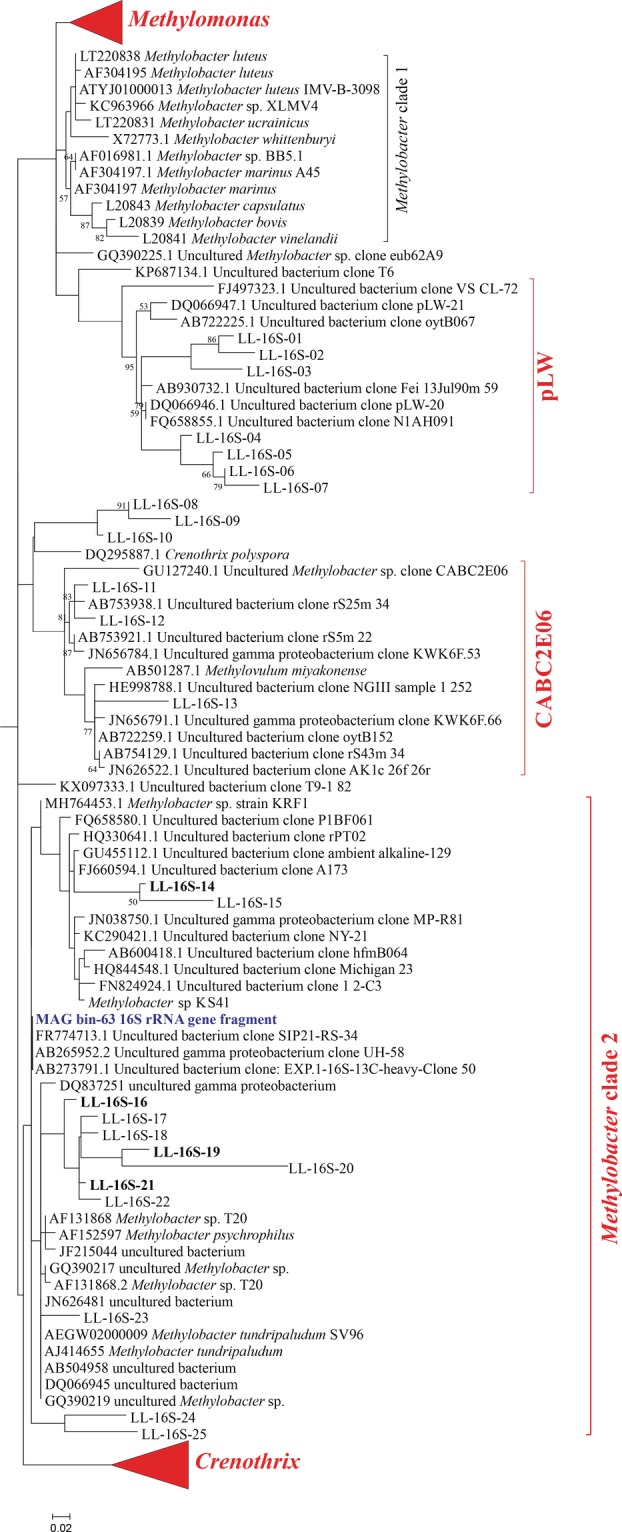
Phylogenetic 16S rRNA gene tree showing 25 representative sequences of the OTUs of the four methanotrophic groups (in red) detected in Lacamas Lake natural waters and incubation experiments (labelled LL‐16S‐followed by a number) and their closest relatives. The most abundant OTUs in the incubations are indicated in bold. OTUs falling in the *Methylobacter* clade 1 cluster were not detected. The relative abundance of the LL‐16S‐ OTUs in each sample are shown in Table S8. The phylogenetic analysis was restricted to the sequence fragment (approximately 290 bp) obtained with the 16S rRNA amplicon sequencing analysis. Maximum likelihood estimation was performed using the General Time Reversible model.

**Figure 4:**
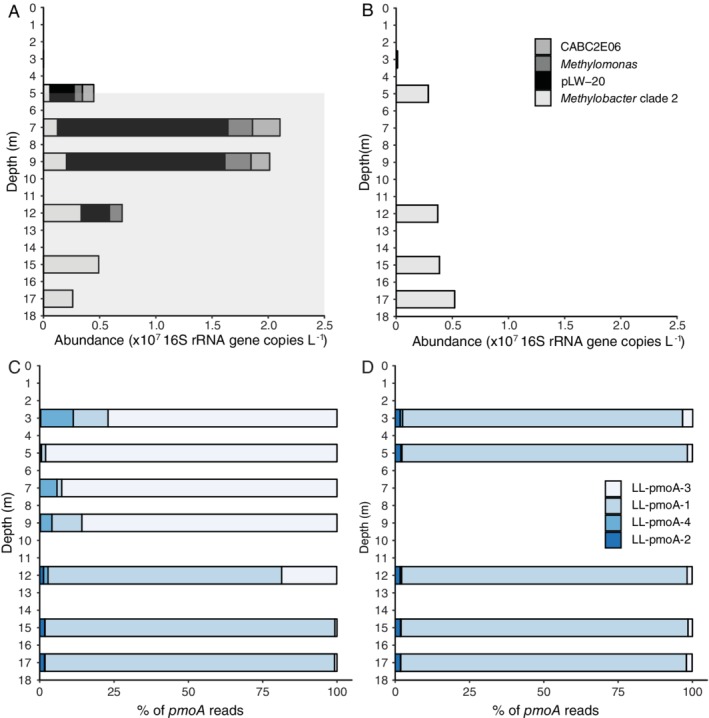
Abundance and composition of the methanotrophic community in the Lacamas Lake water column in summer and winter. (A, B) The absolute abundance of the most important methanotrophic community members, as detected by 16S rRNA gene amplicon sequencing combined with qPCR analysis using the same primers. (C, D) The relative abundance of the detected *pmoA* sequences. The taxonomic assignment of the 16S rRNA gene and *pmoA* OTUs is specified in Figs 3 and 5 respectively. The grey area of (A) indicates the anoxic hypolimnion. Details are given in Table S2 and S3.

We screened the operational taxonomic units (OTUs) obtained by 16S rRNA gene amplicon sequencing for methanotrophic taxa and applied a minimum threshold of 0.1% of the total reads in a given sample. The detected OTUs include members of the Methylococcales, specifically of *Methylomonas*, *Methylobacter* clade 2 (including the cultivated *Methylobacter tundripaludum* and *M*. *psychrophilus*, as described in Smith *et al*., 2018), Crenotrichaceae (*Crenothrix*) and the pLW and CABC2E06 groups (Fig. 3). The eight *Methylobacter* clade 2 OTUs were all closely related (i.e., > 96% identical) to that of *M*. *tundripaludum* (Fig. 3). Total methanotroph abundance as estimated from the 16S rRNA copies was substantially higher in summer than in winter (Fig. 4; Table S2). In summer, methanotrophs were detected at all depths analysed, except at 3 m (Fig. 4A). Both their diversity and their relative abundance were highest at 7–9 m (Fig. 4A); methanotrophs represented 5% of all 16S rRNA gene reads at 7 m (Table S2), with the Methylococcales pLW group being the most abundant (3.3%, 1.5 × 10^7^ 16S rRNA gene copies L^−1^). In the deep anoxic water column, at > 12 m, sequences affiliated to the *Methylobacter* clade 2 were the most abundant (0.3–0.5 × 10^7^ 16S rRNA gene copies L^−1^; Fig. 4A). No methane oxidizing archaea (ANME) were detected. In the oxygen‐rich water column in winter a different picture emerged with only the *Methylobacter* clade 2 present throughout the water column, except at 15 m (Fig. 4B; Table S2).

**Figure 5:**
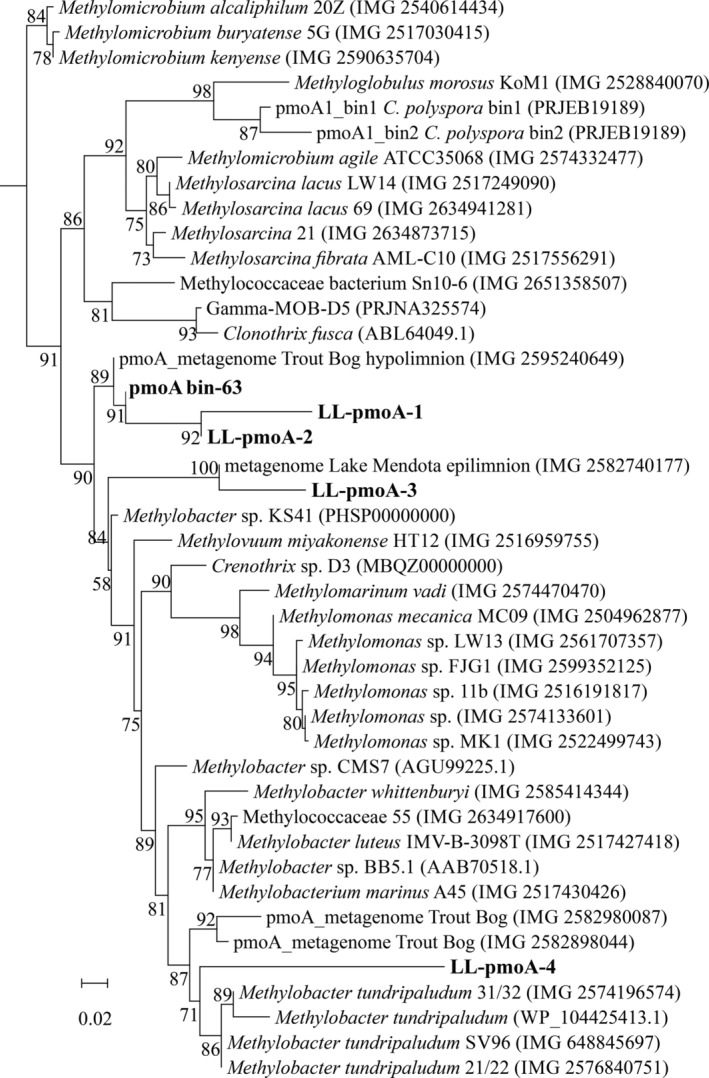
Maximum likelihood phylogenetic tree of PmoA protein sequences (200 amino fragment) obtained with the *pmoA* amplicon sequencing assay (see *Experimental Procedures* for details). The main OTU sequences of Lacamas Lake (LL‐pmoA) as well as the MAG bin‐63 sequence are indicated in bold. Model of protein evolution was LG plus gamma distribution and invariant site, LG+G+I. The scale bar represents number of substitutions per site. Branch support was calculated with the approximate likelihood ratio test (aLRT) and values (%) are indicated on the branches. IMG gene ID number or NCBI accession number are indicated between parentheses.

Functional gene (i.e., *pmo*A) amplicon analysis indicated the presence of four OTUs, of which two dominate (Fig. 4C and D). Their identity was revealed by comparison with available PmoA sequences in databases (Fig. 5). In summer, the upper part of the water column was dominated by the LL‐PmoA OTU LL‐pmoA‐3 (Fig. 4C), which is closely (85%) related to a PmoA coding sequence from a metagenome from the epilimnion of Lake Mendota, and 77–78% identical to that of *Methylobacter* sp. KS41, derived from a metagenome from an acid forest soil enrichment culture. The deeper part of the summer water column was dominated by OTU LL‐pmoA‐1 (Fig. 4C), which also dominated the entire water column in winter (Fig. 4D). The most closely related PmoA sequences are those from a metagenome from the hypolimnion of Lake Trout Bog, and from *Methylobacter* sp. KS41. Closely related PmoA sequences of cultured relatives of both OTU LL‐pmoA‐1 and ‐3 are those of *M*. *tundripaludum* strains (Fig. 5), which fall in *Methylobacter* clade 2 (Fig. 3).

### *Identity and abundance of methanotrophs in incubation experiments*

Anoxic water at 12 m was sampled for summer incubation experiments, based on its high methane concentration (100 μM) and its position about 6 m below the oxycline and about 6 m above the sediment–water interface, which limits potential contamination with sediment or oxycline particles and microbes. Incubations for 72 h with nitrate or sulfate but no added methane were performed to study the effect of electron acceptor availability on the methane oxidation rate and methanotrophic community. The relative abundance of methanotrophs increased from 1.5% of the total 16S rRNA gene reads in the natural water column up to 17.7%, 21.1% and 22.4%, for control incubations and incubations supplied with nitrate or sulfate respectively (Table S2). Sequences affiliated to the *Methylobacter* clade 2 were the most abundant in these incubations (approximately 12% of the total 16S rRNA gene reads), with the OTU sequences LL‐16S‐16 and LL‐16S‐19 (Fig. 3) being the most abundant ones (> 9.5% each of the total sequences classified as *Methylobacter* clade 2) in the incubations supplemented with nitrate. In the summer incubations, nitrate addition slightly stimulated the abundance of PmoA OTUs LL‐pmoA‐4 and LL‐pmoA‐2 (12% and 3.2%, respectively; Fig. S2), which were of minor abundance in the natural water column (1.5% and 1.3%, respectively; Fig. 4C). The addition of sulfate mimicked the conditions in the deeper water column (i.e., > 12 m) and led to strong dominance of the sequence of the OTU LL‐pmoA‐1 (Fig. 4; Fig. S2).

**Figure 6:**
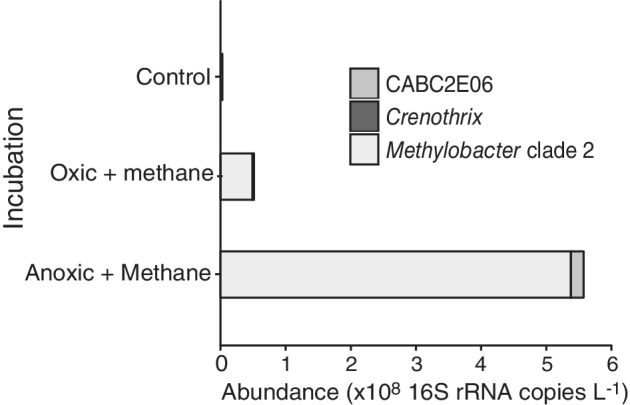
The abundance of methanotrophic community members (as detected by 16S rRNA gene amplicon sequencing) in the winter incubation experiments performed with water from 12 m. Data are provided in Table S2. The OTU LL‐pmoA‐1 dominated all three experiments (Fig. S2).

In winter, when the water column was oxic and depleted of methane, the addition of methane to water from 12 m depth resulted in an increase in the relative abundance of *Methylobacter* clade 2 sequences from 0.7% to 7% of the total 16S rRNA gene reads, corresponding to a slight increase in the absolute abundance (Fig. 6). In addition, incubations were performed under artificially induced anoxic, methane‐rich (194 μM) conditions, mimicking the bottom waters of the stratified lake during summer. This induced a more than 100‐fold increase of the estimated absolute abundance of the *Methylobacter* clade 2 species (Fig. 6), resulting in a major increase in the relative abundance of *Methylobacter* clade 2 from 0.7% of all detected OTUs (control) to 35.4% with OTU LL‐16S‐16 as the most abundant one (i.e., 49% of the sequences classified as *Methylobacter* clade 2; Fig. 3). This corresponds to a doubling time of 9.7 h and, when assuming all methane oxidation is performed by this group, a methane oxidation rate of 0.05 pM cell^−1^ day^−1^. The doubling time of the methanotrophs in the not‐amended incubations and the methane amended incubations was lower (i.e., 63.2 h and 18.6 h respectively). In the anoxic incubations, several other methanotrophs were also detected, although in much lower relative abundances than *Methylobacter* clade 2 species (0.1–1%; Fig. 6). Similar results were observed in incubations with water of 17 m depth although the absolute abundances in the incubations were lower (Fig. S3). The *pmo*A gene amplicon analysis showed the LL‐pmoA‐1 OTU remained dominant in all winter incubations (Fig. S2), as it was in the winter water column (Fig. 4D).

### *Methylobacter species metagenomic analysis*

The winter incubation under anoxic conditions and added methane resulted in a methanotrophic enrichment with members of the *Methylobacter* clade 2 representing more than a third (35.4%) of all 16S rRNA gene reads. Sequencing following a metagenomic approach resulted in several metagenome‐assembled genomes (MAGs), including three MAGs (i.e., bin‐19, ‐37 and ‐63) affiliated with the Methylococcales (Table S4, Supporting Information File S1). MAG bin‐63 was of high quality (96.3% completeness, 0.74% contamination, 0% strain heterogeneity; Table S4) and had the highest average abundance (based on the average coverage of each contig included in the bin; see *Experimental Procedures* for details) of all MAGs obtained. Phylogeny of 34 concatenated marker genes following the method of Dombrowski and colleagues (2018) and GTDB‐Tk analyses revealed that the three obtained MAGs of methanotrophs were most closely affiliated with *Methylobacter* sp. KS41 of the *Methylobacter* clade 2 (Fig. S5). The affiliation of the MAGs was further supported by guanine‐cytosine (GC) coverage plots indicating that the MAG bin‐63 was affiliated to the family Methylomonadaceae (Fig. S6). Furthermore, a phylogenetic analysis of the 16S rRNA gene of the MAG bin‐63, restricted to the 305 bp 16S rRNA gene fragment used for the amplicon sequencing analysis, revealed that it was closely related (average 98%) to the 16S rRNA gene sequences of the *Methylobacter* clade 2 (Fig. 3). Surprisingly, this 16S rRNA gene fragment was not completely identical to the most abundant OTUs (e.g., OTU sequence LL‐16S‐16) of both the anoxic methane‐supplemented winter and nitrate‐supplemented summer incubations (Fig. 3). This may be due to miss‐assembly typically encountered in the SSU rRNA gene (Miller, 2013; Yuan *et al*., 2015) or to potential sequencing mistakes in the amplicon sequencing assay.

The PmoA protein sequence of the MAG bin‐63 was 95.5% identical to the PmoA protein sequence of *Methylobacter* sp. KS41 of the *Methylobacter* clade 2 (see Fig. 3) but the phylogeny is not well supported (58% bootstrap support) similarly to what was observed previously by Nguyen and colleagues (2018). OTU LL‐pmoA‐1 is not identical to the *pmo*A gene coding sequence retrieved from the MAG bin‐63, but as this OTU represented 97% of the total *pmo*A gene sequences in the sample from which the metagenome was obtained, it is likely that both the *pmoA* of MAG bin‐63 and the OTU LL‐pmoA‐1 are the same. MAG bin‐63 was by far the most abundant bin retrieved from the enrichment and is therefore unlikely related to a species that represented less than 3% of the *pmoA* gene sequences.

Based on the highest average abundance (Table S4) of the MAG bin‐63, its closest homology to the representative 16S rRNA gene sequences affiliated with the *Methylobacter* clade 2 in that given sample (Fig. 3), and the phylogenetic placement of the MAGs reported in this study, we conclude that the MAG bin‐63 is very likely affiliated to the *Methylobacter* clade 2 and representative for the *Methylobacter* species that in summer predominantly resides in the anoxic hypolimnion of Lacamas Lake and in winter is present throughout the oxic water column albeit in lower abundances.

### *Genome‐inferred metabolism of the Methylobacter MAG bin‐63*

**Figure 7:**
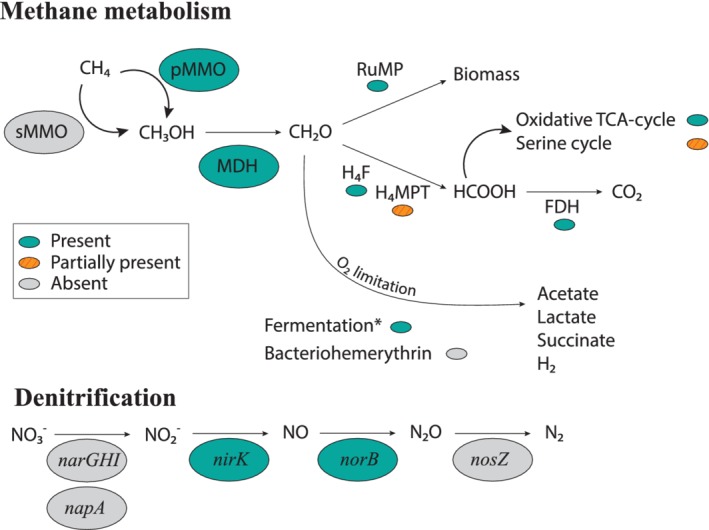
Metabolic pathways of methane oxidation and denitrification in the predominant *Methylobacter* species present in Lacamas Lake as inferred from the presence of encoding genes in MAG bin‐63. Enzymes/pathways indicated in green were encoded by the genome, orange‐streaked indicates the presence of an incomplete pathway. Grey pathways were not detected. The asterisk indicates the full fermentation pathway as shown in Fig. S4. FDH, formate dehydrogenase; H4F, methylene tetrahydrofolate pathway; H_4_MPT, tetrahydromethanopterin pathway; MDH, methanol dehydrogenase; RuMP, ribulose monophosphate pathway.

Due to its high quality and highest average abundance compared to the other MAGs, we will focus here on the description of the metabolic potential of MAG bin‐63. All genes encoding for particulate methane monooxygenase (pMMO) are present and organized in the *pmo*CAB operon (see Supporting Information File S1), uniquely found in type Ia methanotrophs (Trotsenko and Murrell, 2008; Villada *et al*., 2019). The sequence‐divergent particulate monooxygenase (Pxm; Tavormina *et al*., 2011; Knief, 2015) is absent based on a blast search with the PxmA sequence of *M*. *tundripaludum*. Homologues of the soluble methane monooxygenase (sMMO) are also absent based on a blast search with the *mmo*X gene of *C*. *polyspora*. The gene coding for methanol dehydrogenase is present (Fig. 7).

Genes for a complete RuMP pathway for C‐1 assimilation from formaldehyde are present, as well as those for the oxidative tricarboxylic acid (TCA) cycle, while the serine cycle is incomplete (Fig. 7). Genes involved in H_4_folate (*mtd*A‐*fch*), membrane‐associated quinoprotein formaldehyde dehydrogenase (*ald*) and formate oxidation (*fds*) are identified (Fig. 7; Supporting Information File S1). Genes for the H_4_MTP‐linked C‐1 transfer are present with the exception of the methylene‐tetrahydromethanopterine dehydrogenase (*mtd*B, see Fig. 7). MAG bin‐63 has the genetic potential to perform mixed‐acid fermentation from pyruvate to succinate, and from pyruvate to acetate via acetyl‐CoA (Fig. S4). Genes for the formation of lactate from pyruvate are lacking, as well as a complete pathway from pyruvate to acetate via acetylphosphate (Fig. S4).

The MAG also includes the genes of the bidirectional NAD‐reducing hydrogenase Hox system that could either produce hydrogen or use H_2_ as electron donor (see Fig. S4). We did not find genes coding for the O_2_‐carrier (bacterio)hemerythrin, which has previously been suggested to be involved in O_2_ scavenging or in shuttling O_2_ directly to the PmoA enzyme complex (Chen *et al*., 2012).

Two types of aerobic respiratory chain complexes are encoded (Fig. S4): the proton‐pumping type I NADH dehydrogenase (NDH‐1, complex I; encoded by the *nuoA‐N* operon) and the sodium‐pumping NADH dehydrogenase (Na^+^‐NQR). The non‐proton pumping type II NADH dehydrogenase (NDH‐2; *ndh* and *ndh*A) was not detected. Genes coding for the terminal reductases of the cytochrome c oxidase complex (i.e., *cyo*E, *cox*CAB) and the cytochrome *bd* oxidase complex (also known as quinol reductase *bd* terminal reductase or high‐affinity cytochrome *bd* ubiquinol oxidase, *cyd*AB; Fig. S4) were also detected. The denitrification pathway was incomplete: *nirK* and *norB* genes, involved in dissimilatory nitrate reduction, are present (Fig. 7, Supporting Information File S1), but the dissimilatory nitrate reductase (*nar*G) and nitrous oxide reductase (*nosZ)* genes are absent.

## Discussion

Both the high methane oxidation rates in the anoxic water column as well as the results of the anoxic incubations suggest that AOM takes place in Lacamas Lake. In previous studies, freshwater methane oxidation under anoxic conditions has been observed to be performed by ANME (Eller *et al*., 2005; Zigah *et al*., 2015), by methane‐oxidizing bacteria working in close cooperation with photosynthetic oxygen producers (Oswald *et al*., 2015), or by bacteria of the *Ca*. M. oxyfera, which are providing their own oxygen via a nitrite‐reduction pathway (Ettwig *et al*., 2010). None of these three mechanisms is likely to be responsible for methane oxidation in the hypolimnion of Lacamas Lake. No more than 0.2% of all 16S rRNA reads could be assigned to archaea, and none of the known ANME clades were detected. Although primer bias may decrease the number of reads assigned to archaea, the absence of ANME is not surprising, given the oxic/anoxic cycles in Lacamas Lake and the intolerance of ANME for oxygen. Although strict anaerobes could potentially survive in anoxic sediment layers during winter oxygenation, it is unlikely they are capable of rapidly occupying the anoxic niche when stratification occurs, especially considering their slow growth rates (ANME doubling times of 2–7 months; Nauhaus *et al*., 2007; Scheller *et al*., 2016). Methane oxidation fuelled by photosynthesis in the hypolimnion would require deep light penetration, whereas Lacamas Lake secchi disc depth was < 2 m at the time of sampling, making this option unlikely. Likewise, *Ca*. M. oxyfera or other NC10‐related 16S rRNA gene sequences were not detected in Lacamas Lake.

Methanotrophs, such as *Methylomonas denitrificans*, *C*. *polyspora* or *M*. *tundripaludum*, who couple methane oxidation to nitrite or nitrate reduction (Kits *et al*., 2015a,b; Oswald *et al*., 2017; Rissanen *et al*., 2018), are also potential candidates to perform methane oxidation in the anoxic water column. However, in our study, members of the *Methylobacter* clade 2 were identified as the dominant methanotrophs in the anoxic water column (12–17 m) during summer, in the summer water column incubations, at all depths of the oxic water column in winter, and in the anoxic winter incubations (Fig. 4C and D), as observed by the 16S rRNA gene (Fig. 3) and PmoA coding gene (Fig. 5) sequencing. This indicates that the *Methylobacter* clade 2 species detected in our study thrive not only under oxic conditions but also in anoxic environments, suggesting that this species is also capable of AOM like some other methanotrophs. The strongest evidence for this conclusion comes from the anoxic winter incubation experiment that resulted in a strong (39%) enrichment of a *Methylobacter* clade 2 species (Fig. 4).

Although low amounts of oxygen could have been present at the start of the anoxic incubation experiments, due to oxygen contamination during sampling or handling, we can rule this out as a driver of methane oxidation for several reasons. Firstly, the observed methane decrease over time was highly linear (*R* ^2^ of 0.999) and did not show an increased oxidation in the first 6 h, which would have been expected if oxygen contamination would have stimulated methane oxidation rates (Table S1). Secondly, the addition of oxygen to the summer incubation experiment with anoxic water from 12 m diminished methane oxidation (Fig. S1), showing that high oxygen concentrations inhibited methane oxidation, as was previously observed by Thottathil and colleagues (2019). Thirdly, De Brabandere and colleagues (2012) estimated the oxygen introduction by leakage from the oxygen‐containing butyl caps of exetainers at about 300–400 nM l^−1^. Given the 2:1 stoichiometry of aerobic methane oxidation, oxygen contamination could thus only have accounted for the first 0.15–0.2 μM oxidized in the exetainers, which is only 0.3% of the total oxidized methane. The stoppers used were double waded and should therefore give maximum protection against leakage. Oswald and colleagues (2016) estimated the maximum intrusion through butyl stoppers in serum bottles to be 13 nM day^−1^. This could account for only 0.02% of the methane oxidation. Hence, although we cannot rule out the presence of traces of oxygen, the quantity of oxygen is simply not high enough to explain the amount of methane that is oxidized.

Several methanotrophs are known to contain genes encoding for parts of the nitrate reduction pathway, which has previously been shown to be coupled to methane oxidation under anoxic conditions (Smith *et al*., 2018). Specifically, the genomes of some *Methylobacter* species encode a complete nitrate reduction pathway up to N_2_O, possibly allowing them to use nitrate as a terminal electron acceptor when oxygen is limiting (Smith *et al*., 2018). However, other *Methylobacter* species only contain the *nirS*/*nirK* and *norB* genes, but lack the *narG* and *napA* genes for nitrate reduction, making them unable to perform complete denitrification (Svenning *et al*., 2011; Smith *et al*., 2018). *Methylobacter* species and closely related methanotrophs have previously been found to thrive in anoxic environments where methane oxidation could not be explained by the presence of known electron acceptors or pathways (Biderre‐Petit *et al*., 2011; Blees *et al*., 2014; Martinez‐Cruz *et al*., 2017). The *Methylobacter* species detected in our study are not 100% identical to the known nitrate‐reducing species *M*. *tundripaludum* (Figs 3 and 5). The genome of the MAG bin‐63 that reflects the abundant *Methylobacter* species in Lacamas Lake lacked the nitrate reductase coding gene (*nar*G) involved in the dissimilatory reduction of nitrate (Fig. 7). This suggests that the dominant Lacamas Lake *Methylobacter* species cannot perform nitrate reduction itself, unless it utilizes reduction pathways that are up to this point unknown or incorrectly classified. If this species cannot perform nitrate reduction, it will need to reduce another electron acceptor to be able to perform methane oxidation. Potentially, it could use nitrite as an electron acceptor, as its genome contains the nitrite reductase gene, *nirK*, and the nitric‐oxide reductase gene *norB*. Nitrate, but not nitrite, was provided to the experiments, but a non‐methanotrophic organism could have converted the nitrate to nitrite before being used by the methanotrophs. Nitrate reduction was not measured in our experiments, so we can only speculate about these processes occurring. Nitrite reduction has been observed in *Methylomicrobium album* BG8 by Kits and colleagues (2015a), but not in any other methanotroph up to this point. No metabolic pathway involved in sulfate reduction could either be identified in the genome of the MAG bin‐63, even though the addition of sulfate stimulated methane oxidation in the anoxic incubation experiments (Fig. 2).

The question of how this *Methylobacter* species can thrive under anoxic conditions (fivefold abundance and fourfold oxidation rate increase with respect to oxic conditions, Figs 2B and 6), therefore, remains fascinating but difficult to answer at this stage. The genome of MAG bin‐63 included a complete RuMP pathway for C‐1 assimilation from methane‐derived formaldehyde, and a complete mixed‐acid fermentation pathway to fumarate and acetate, indicating both high and low‐oxygen adapted metabolism. The presence of the high‐affinity cytochrome *bd* oxidase complex, the NADH‐quinone oxidoreductase (nqr) and hydrogen dehydrogenase genes (Fig. S4) also support this hypothesis of low‐oxygen adaptation. Fermentation‐based methanotrophy has been detected before and it has been hypothesized that under low oxygen conditions, a metabolic switch to fermentation can be induced (Morinaga *et al*., 1979; Roslev and King, 1995; Kalyuzhnaya *et al*., 2013; Gilman *et al*., 2017), which may also occur in the *Methylobacter* species detected in Lacamas lake. Fermentation‐based methanotrophy has, however, been shown to lead to low biomass production and methane turnover rate in a culture study of *Methylomicrobium alcaliphilum* (Kalyuzhnaya *et al*., 2013), while biomass production by the *Methylobacter* in our experiments was exceptionally high, with doubling times of < 10 h under anoxic conditions (Fig. 6). In addition, methane oxidation rates in Lacamas Lake's anoxic hypolimnion were higher than those reported in comparable lakes with a similar or lower methane concentration (Eller *et al*., 2005; Schubert *et al*., 2010; Blees *et al*., 2014). This raises considerable concerns whether this *Methylobacter* species can rely solely on a fermentation‐based metabolism for methane oxidation.

An alternative explanation could be the existence of a metabolic syntrophy between the methanotrophs converting methane into organic compounds (e.g., acetate, fumarate) and hydrogen, and microorganisms able to metabolize these excreted compounds (see Yu and Chistoserdova 2017 for a review). These partner organisms could stimulate methane oxidation by *Methylobacter* cells, and therefore the biomass production, by consumption of growth‐limiting excreted compounds.

Here, we identify the potential syntrophic partner of the *Methylobacter* species by analysing which microorganisms were high in relative abundance or increased in relative abundance together with the increase of the *Methylobacter* species. Sequences affiliated with the order Burkholderiales (*Betaproteobacteria*) occur abundantly in Lacamas Lake (up to 16.9% and 35.1% in the summer and winter water column, respectively; Table S5). Members of these groups have been described as being able to assimilate succinate, while using nitrate as an electron acceptor (Saito *et al*., 2008). Bacteria of the family *Methylophilaceae* (*Betaproteobacteria*) also showed an increased abundance in the incubations with the highest methane oxidation rates (Fig. S5). They have often been detected in co‐occurrence with methanotrophs, and have been shown to use reaction products of methanotrophy (Yu and Chistoserdova, 2017), coupling methanol oxidation to nitrate reduction (Kalyuzhnaya *et al*., 2012). It is, however, unknown whether there is an advantage for the methanotrophs in this relationship. Furthermore, sequences closely related to the order Rhodocyclales were highly abundant in the anoxic summer water column and incubations, especially the genus *Sulfuritalea* (7.2–12.9%, Table S5). *Sulfuritalea hydrogenivorans* was isolated by Kojima and Fukui (2011) from the water column of a stratified lake and described as a facultative anaerobe, capable of oxidizing thiosulfate and hydrogen, using nitrate as an electron acceptor. Although we stress that the co‐occurrence of these species cannot be used as a proof of a relationship and that the fermentation potential of the *Methylobacter* detected here is purely based on genome‐inferred information, the co‐occurrence of *Methylobacter* and Burkholderiales, *Methylophilaceae* and *Sulfuritalea* species is intriguing and would be a good target for further research, exploring whether the observed stimulation by nitrate in our incubation experiments could be indirect, via partner organisms. The stimulation of methane oxidation in incubation experiments with added sulfate could work similarly, although no potential partner organisms could be identified at this stage.

## Conclusions

*Methylobacter* species have been found in many low‐oxygen and anoxic zones in stratified lakes, similar to Lacamas Lake, but the electron acceptor used for methane oxidation remained unclear. Although the addition of nitrate and sulfate was found to stimulate methane oxidation in Lacamas Lake, the absence of a complete nitrate reduction pathway in the genome of the Lacamas lake *Methylobacter* species means the exact mechanism mediating this oxygen‐limited methane oxidation remains unknown. No pathway involved in sulfate reduction could be identified either. Possibly, another member of the microbial community, capable of nitrate reduction, could provide the detected *Methylobacter* with nitrite, which could then be reduced to N_2_O, coupling methane oxidation to nitrite reduction. Consortia of methanotrophs and partner organisms are well known from marine settings (i.e., sulfate‐reducing bacteria and ANME; Boetius *et al*., 2000), and have been suggested to also occur in lakes (Oshkin *et al*., 2014; Hernandez *et al*., 2015; Yu and Chistoserdova, 2017). The apparent lack of electron acceptors and genomic pathways that can explain methane oxidation by *Methylobacter* cells alone suggest that such a lacustrine consortium may exist, but clearly more research is required to explore the involved organisms and metabolic pathways.

Mixed‐acid fermentation, as suggested for the detected *Methylobacter* species based on its genome, could be widespread in bacterial methanotrophs. The assumption that the key product of methane oxidation is always CO_2_ might, therefore, need to be reconsidered, as fermentation products such as lactate, succinate and acetate could be a substantial sink for methane‐derived carbon in oxygen‐limited systems. Research on the genomic capacities for mixed‐acid fermentation and the expression of these genes should be performed, as well as studies that focus on measuring the reduction of electron acceptors by methanotrophs and other possibly involved microorganisms, in order to find the link between electron acceptor reduction and methane oxidation rates.

## Experimental procedures

### *Site description*

Lacamas Lake is a temperate zone lake in Washington, USA (45.62 N, 122.43 W) with an average and maximum depth of 7.8 and 19.8 m respectively. In 1938, a dam was built to enlarge and deepen the existing lake to its current size of 1.3 km^2^. Lacamas Lake is an Environmental Protection Agency 303[d] listed hyper‐eutrophic system. It is monomictic, with thermal stratification established in May and a turnover period from October to December during which the oxycline deepens and weakens gradually.

### *Sample collection*

Samples were taken from one sampling location in the centre of the lake (water depth 17.8 m) on 23–31 August 2016 and 6–10 February 2017. The depth of the oxycline was determined using a Hydrolab DS5X sonde (Hach, Loveland, USA) with sensors for conductivity, temperature, dissolved oxygen (Clark cell) and pH. Water samples were taken using a VanDorn sampler or Niskin bottle. To determine nutrient (${\mathrm{NO}}_{3}^{-}$, ${\mathrm{NO}}_{2}^{-}$, ${\mathrm{SO}}_{4}^{2-}$) concentrations, bulk water samples were subsampled in the field, treated as described in Table S6 and kept on ice until they could be frozen and stored at −20°C until further analysis using a Technicon TRAACS 800 auto‐analyser. Samples for the measurement of methane oxidation rates were taken directly into double waded 12 mL exetainers (Labco, High Wycombe, UK), or in glass bottles (Neubor, San Vito al Tagliamento Pordenone, Italy), overflowing the vessels with three times the sample volume before filling the vessels without any headspace. Water for DNA sampling was stored in plastic carboys or jugs and kept shielded from temperature fluctuations and light with emergency insulation blankets, to be filtered later in the laboratory. In Table S6, an overview of the sampling depths, sampling purposes and handling is provided.

### *Determination of methane oxidation rates*

In order to determine natural net methane oxidation rates, samples were directly sampled into double waded 12 mL exetainers (Labco, High Wycombe, UK). For each depth, 16 exetainers were filled, of which 4 were treated with approximately 50 μl of saturated zinc chloride solution (ZnCl_2_), added immediately to terminate any biological activity. Exetainers were kept in coolers at lake temperature until back in the lab, where they were stored in the dark, in water baths within ±2°C of in situ temperature. Approximately 50 μl of saturated ZnCl_2_ solution was injected into four exetainers, functioning as biological replicates, per time point, at 6, 12 and 24 h after the start of the incubation.

To determine the net methane oxidation rate in summer samples with added electron acceptors, samples were taken in a slightly different way. In the field, samples of 12 m depth were taken into glass bottles (315 mL with rubber stopper), which already contained ~0.005 g NaNO_3_, ~0.1 g Na_2_SO_4_ or ~0.004 g of commercially available humic substances (Sigma Aldrich). Bottles were not overflown but were carefully filled without any headspace. Oxygen addition was tested by leaving a 10 mL air headspace in the bottle, which was shaken to dissolve the oxygen into the water. In the lab, using the method of Holtappels and colleagues (2011) nutrient samples and 16 exetainers were filled, 2–8 h after sample collection. These were further treated in the same way as the natural methane oxidation rate samples.

During winter sampling, exetainers for natural net methane oxidation rate and methane‐added incubations were taken similarly to the summer natural methane oxidation rate samples. Samples for the anoxic incubations were taken in glass bottles (315 mL with rubber stopper), overflowing the vessels with three times the sample volume before filling the vessels without any headspace, bubbled with ultra‐high purity helium for 10–15 min to remove oxygen, and transferred to exetainers using the method of Holtappels and colleagues (2011). We use the term ‘anoxic’ for these experiments, and for experiments with summer hypolimnion water, despite the fact that we cannot be certain that no traces of oxygen were present in the incubation vials. The possible effects of residual oxygen are explored in the *Discussion*. Both the methane‐added and anoxic incubations got methane added by injection through the stopper of the exetainers, to a final concentration of 134–260 μM. Control incubations were left un‐amended and did, therefore, not receive methane, neither were they purged with helium. Four exetainers got approximately 50 μl of saturated ZnCl_2_ solution injected immediately to determine the t_0_ methane concentration. The other exetainers, four biological replicates per treatment, were incubated in the dark in water baths within ±2°C of in situ temperature, and ZnCl_2_ was added 6, 12 and 24 h after the start of the incubation. ZnCl_2_‐poisoned samples were stored upside down at room temperature until further analysis. To measure the methane concentration in the exetainers, 1 mL high purity nitrogen (N_2_) headspace was generated, the exetainers were left to equilibrate for at least 48 h and then measured as technical triplicates on a gas chromatography system including a flame ionization detector. Net methane oxidation rates were determined using linear regression (*p* < 0.05). The addition experiments had final nitrate concentrations approximately 10 times higher than the natural concentrations of the control experiment, or sulfate concentrations 100 times higher than the natural concentrations of the control experiments (Table S1). The concentrations of oxygen, nitrate and sulfate at the end of the experiments were not determined. In the 7 m depth summer incubations, the methane concentration reached zero before the end of the incubations, and only the time points before methane depletion were used. In the 15 m summer control experiment, the concentration increased initially, but decreased linearly from 6 until 24 h. Only this last part of the incubation was used for determination of the methane oxidation rate. To calculate the theoretical amount of methane that could be oxidized with the concentration of electron acceptor in the vial, an 8:3 ratio of NO_3_:CH_4_ and a 1:1 ratio of SO_4_:CH_4_ was assumed, after Segarra and colleagues (2013). The simplification was made that no other processes consumed the oxidizing power of the added electron acceptor.

Per cell methane oxidation rates were calculated by dividing the oxidation rate per litre by the number of cells per litre. The doubling time was calculated with the formula: doubling time = *t*/[3.3*log(*b*/*B*)], with *t* being the time in hours, *b* being the number of cells at *t*_end_ and *B* being the number of cells at *t*_0_.

### *Microbial community sampling and incubations*

For the natural community samples, 3.9 L sample water was filtered over pre‐ashed 0.3 μM glass fibre filters (45 mm diameter, Whatman, Maidstone, UK) within 2–4 h after sample collection. Filters were stored at −20°C until DNA extraction. Water for summer microbial community incubations was collected in 3.9 L jugs. If applicable, salts (NaNO_3_ or Na_2_SO_4_) were added in the same concentrations as those in the methane oxidation rate incubations. Winter microbial community samples were taken in the above‐mentioned glass bottles with rubber stoppers. Anoxic incubations were bubbled with ultrapure helium for 10 min to remove oxygen. The methane‐addition and O_2_ removal experiments got 0.66 mL of 99.99% pure methane gas added per bottle, in a headspace of 12 mL ultrahigh purity helium, leading to a concentration of 150–260 μM methane. To maintain oxic conditions, the oxic winter incubations with and without methane had an air headspace of 12 mL. All microbial community incubations were kept in the dark at ±2°C of in situ temperature and were shaken every 24 h. After 72 h, they were filtered over pre‐ashed 0.3 μM glass fibre filters (Whatman, Maidstone, UK) and stored at −20°C.

### *DNA extraction and 16S rRNA, pmoA gene amplification, analysis and phylogeny*

DNA was extracted from 1/32 to 1/8 part of the filters using the PowerSoil DNA extraction kit (Mo Bio Laboratories, Carlsbad, CA, USA). DNA extracts were stored at minus 80°C until further analysis. The 16S rRNA gene amplicon sequencing and analysis was performed with the general 16S rRNA archaeal and bacteria primer pair 515F and 806RB targeting the V4 region (Caporaso *et al*., 2012) as described in Besseling and colleagues (2018).

A fragment of the *pmo*A gene (approximately 550–583 bp) was targeted for amplicon sequencing by using the forward primer A189 and an equimolar mixture of the reverse primers A682r and Mb661r (Holmes *et al*., 1995; Costello and Lidstrom, 1999). PCR products were gel purified using the QIAquick Gel‐Purification kit (Qiagen), pooled and diluted. Sequencing was performed by the Utrecht Sequencing Facility (Utrecht, The Netherlands), using an Illumina MiSeq sequencing platform. The 16S rRNA gene amplicon sequences were analysed by the Cascabel pipeline (Asbun *et al*., 2019) including quality assessment by FastQC (Andrews, 2010), assembly of the paired‐end reads with Pear (Zhang *et al*., 2014), library demultiplexing, OTU clustering and representative sequence selection (‘longest’ method) by diverse Qiime scripts (Caporaso *et al*., 2010). The OTU clustering algorithm was uclust (Edgar, 2010) with an identity threshold of 97% and assign taxonomy with BLAST (Altschul *et al*., 1990) by using the Silva 128 release as reference database (https://www.arb-silva.de/; Quast *et al*., 2013). Representative methanotrophic sequences were extracted from the data set and the sequences were added to the reference tree of the release 128 of the Silva NR SSU Ref database (http://www.arb-silva.de/; Quast *et al*., 2013) using the ARB software package (Ludwig *et al*., 2004) by means of the ARB Parsimony tool. The 16S rRNA amplicon reads (raw data) have been deposited in the NCBI Sequence Read Archive (SRA) under BioProject number PRJNA524776 (Biosamples SAMN11032793 to SAMN11032810).

The *pmo*A gene amplicon sequences were analysed with the same in‐house pipeline mentioned above for the 16S rRNA gene analysis (including clustering of OTUs at 97%) but performing the taxonomy assignation through BLAST against the NCBI database of non‐redundant nucleotides (NT). The phylogenetic tree was restricted to the about 200 amino acid fragment covered by the *pmo*A gene amplicon sequencing analysis. Representative *pmo*A gene sequences from the amplicon sequencing analysis as well as the *pmo*A gene sequence(s) extracted from the MAGs were added to the phylogenetic tree of compiled *pmo*A gene coding sequences included in Oswald and colleagues (2017). The phylogenetic tree included PmoA protein sequences retrieved from the Integrated Microbial Genomes database (IMG‐ER; Markowitz *et al*., 2009) as indicated in Oswald and colleagues (2017), protein sequences of ‘unusual’ PmoA of *C*. *polyspora* (accession ABC59822–ABC59827; Stoecker *et al*., 2006), partial PmoA of *Clonothrix fusca* (accession ABL64049; Vigliotta *et al*., 2007), as well as the PmoA protein sequences of *Crenothrix* retrieved by Oswald and colleagues (2017). Alignments were performed in MEGA6 (Tamura *et al*., 2013) with Muscle (Edgar, 2004). Models of protein evolution were determined in MEGA6. Maximum likelihood phylogenetic trees were determined using PhyML v.3.0 (Guindon *et al*., 2010). The pmoA reads have been deposited in NCBI, the accession numbers are pending.

### *qPCR 16S rRNA gene*

16S rRNA gene copies were quantified using qPCR with the same primer pair as used for amplicon sequencing (515F, 806RB). The qPCR reaction mixture (25 μL) contained 1 U of Pico Maxx high fidelity DNA polymerase (Stratagene, Agilent Technologies, Santa Clara, CA) 2.5 μL of 10x Pico Maxx PCR buffer, 2.5 μL 2.5 mM of each deoxyribonucleotide triphosphate, 0.5 μL bovine serum albumin (20 mg mL^−1^), 0.02 pM μL^−1^ of primers, 10,000 times diluted SYBR Green® (Invitrogen) (optimized concentration), 0.5 μL of MgCl_2_ (50 mM) and ultrapure sterile water. The cycling conditions for the qPCR reaction were the following: initial denaturation at 98°C for 30 s, 45 cycles of 98°C for 10 s, 56°C for 20 s, followed by a plate read, 72°C for 30 s, 80°C for 25 s. Specificity of the reaction was tested with a gradient melting temperature assay, from 55 to 95°C with a 0.5°C increment for 5 s. The qPCR reactions were performed in triplicate with standard curves from 10^0^ to 10^7^ molecules per microliter. qPCR efficiency for the 16S rRNA quantification was 103.7% with *R* ^2^ = 0.993%, and 109.8% with *R* ^2^ = 0.991. For quantification of microbial groups, we made the assumption that all the microorganisms of the microbial community in Lacamas Lake contained a single 16S rRNA copy in their genome. For analysis purposes, only species with a relative abundance > 0.01% were assumed significant.

### *Metagenome analysis*

Sample collection and DNA extraction of the sample used for metagenome sequencing are described above. DNA of the sample of interest was used to prepare a TruSeq DNA nano library which was further sequenced with Illumina MiSeq 2 × 300 bp generating over 46 million 2 × 300 bp paired‐end reads. Data were analysed with an in‐house pipeline including evaluation of sequence quality with FastQC v.0.11.3 (Andrews, 2010) and removal of the adapters with Trimomatic v.0.35 (Bolger *et al*., 2014). Reads were assembled into contigs using MetaSPAdes v.3.11.1 (Nurk *et al*., 2017), and evaluation of the quality of the assembly with Quast v.4.5 (Gurevich *et al*., 2013). The assembled reads were mapped back against the raw data with BWA‐MEM v.0.7.12‐r1039 (Gurevich *et al*., 2013). Contigs were binned into draft genome sequences based on tetra‐nucleotide frequencies with MetaBAT v.2.11.1, which was also used for estimation of the contig depth, coverage and statistics with the script jgi_summarize_bam_contig_depths (Kang *et al*., 2015). Quality of the MAGs was assessed using CheckM v1.0.7 running the lineage‐specific workflow (Parks *et al*., 2015). MAGs were annotated with Prokka v1.12 (Seemann, 2014) and by the Rapid annotation using subsystem technology (RAST) pipeline v2.0 (Aziz *et al*., 2008). The annotation of key metabolic pathways was refined manually. In order to classify the MAGs according to their relative abundance in the sequenced sample, MetaBAT was run again by using the abundance estimation (total average depth, average abundance or also called average coverage of each contig included in the bin) generated by MetaSPAdes and checked again with CheckM. The phylogenetic placement of the MAGs with higher average abundance was determined by using Phylosift (v. 1.0.1, Darling *et al*. (2014)) extracting 34 marker genes as described in Dombrowski and colleagues (2018) (Table S7). The MAGs were then compared to all publicly available genomes from the same taxonomic group. Additionally, GTDB‐Tk (v0.3.2; http://gtdb.ecogenomic.org) and GC coverage plots (gc cov.pl script included in https://www.michaelgerth.net/resources.html) were also used to assign the taxonomic classification of the MAGs (see Table S4, Fig. S6).

The metagenome of the winter water sample incubation under anoxic conditions and supplementation of methane (Lac_W_12m_72h) is available in NCBI under project number PRJNA524776, biosample SAMN11032804. The sequence of the MAGs bin‐19, bin‐37 and bin‐63 are deposited in Integrated Microbial Genomes and Microbiomes (IMG; https://img.jgi.doe.gov/) under submission ID numbers 204587, 204588 and 204589 respectively.

### *Identification of specific coding genes and phylogenetic analyses*

Specific coding genes of the methane metabolism as well as coding genes involved in the potential aerobic and anaerobic respiration in MAG bin‐63 that failed to be annotated by Prokka and RAST were further investigated by performing protein blast searches with curated protein sequence reference data sets.

## Supporting information


**Appendix S1.** Supporting Information.

**Appendix S2.** Supporting Information.
