# Supplementary material for: Methane oxidation in anoxic lake water stimulated by nitrate and sulfate addition
Source: Environ Microbiol. 2020 Jan 1;22(2):766–82. doi: 10.1111/1462-2920.14886 (PMC7027835; doi:10.1111/1462-2920.14886)
Supplement: Supplementary file 1 — Appendix S1. Supporting Information. [file EMI-22-766-s001.pdf]

Supplemental Information with “Methane oxidation in anoxic lake water stimulated by nitrate and sulfate addition” (van Grinsven et al.)

Fig. S1. Methane concentration over time in the incubation experiment with 12 m depth summer samples, with oxygen (grey triangles) or humic substance (orange circles) added. No significant change in methane concentration over time was observed, the R^2 of the linear regression analysis was 0.04 for the oxygen addition experiment and 0.03 for the experiment with the addition of humic substances.

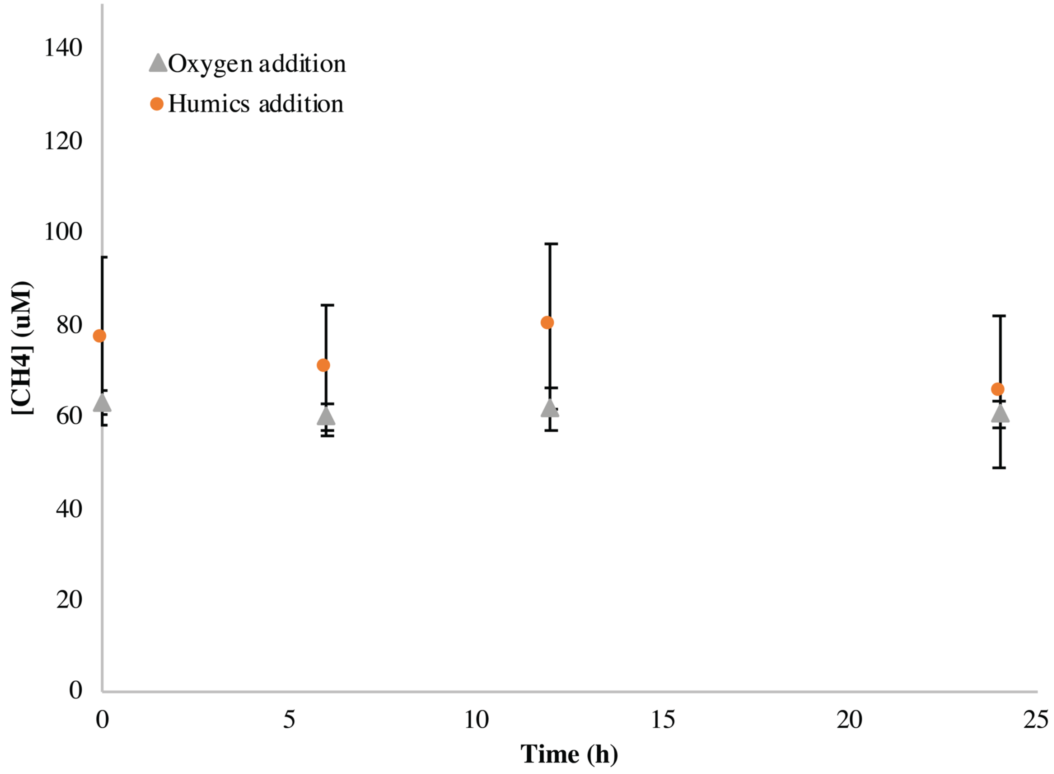

Fig. S2. PmoA distribution of incubation experiments in summer (A) and winter (B). Numbers are provided in Table S3.

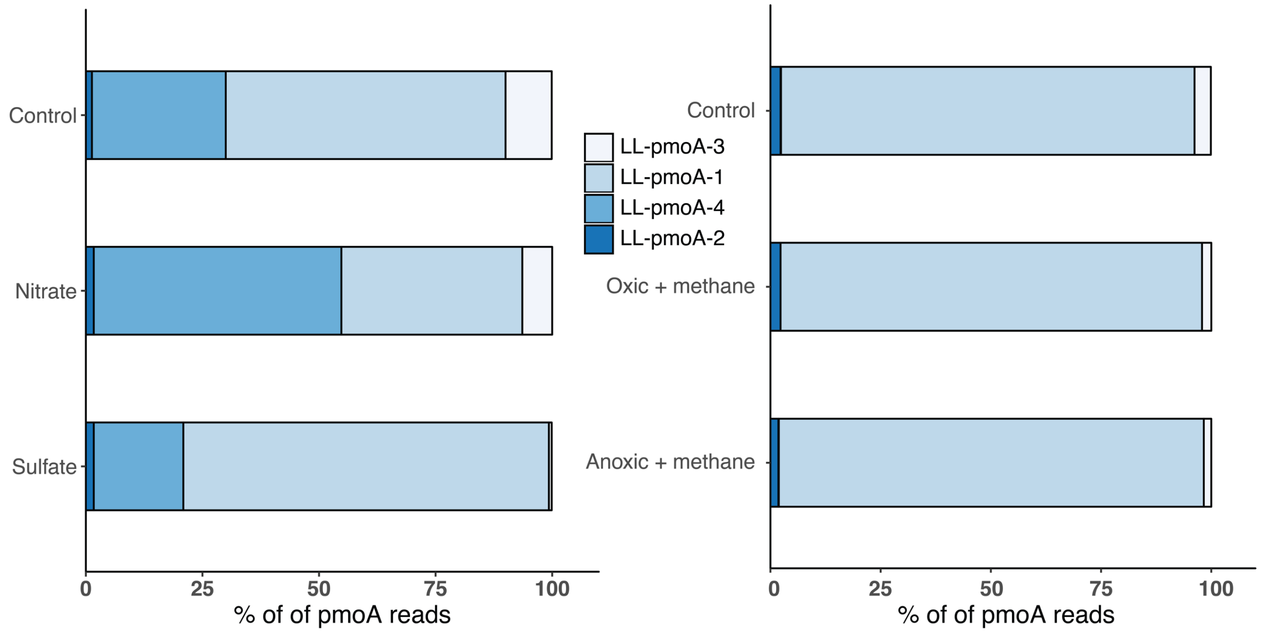

Fig. S3. The abundance of methanotrophic community members (as detected by 16S rRNA gene amplicon sequencing) in the winter incubation experiments performed with water from 17 m depth.

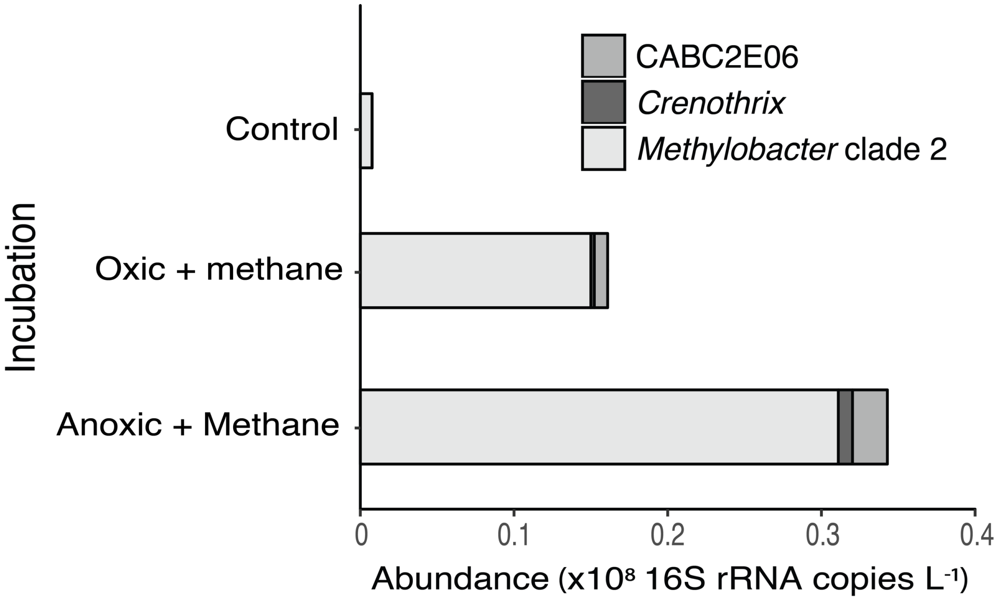

Fig. S4. Genome-inferred metabolic pathways of MAG bin-63. Pathways indicated in green were detected, sequences of grey pathways were lacking. Genes were indicated where possible; numbers refer to EC database numbers. For details, see Supplementary File S1.

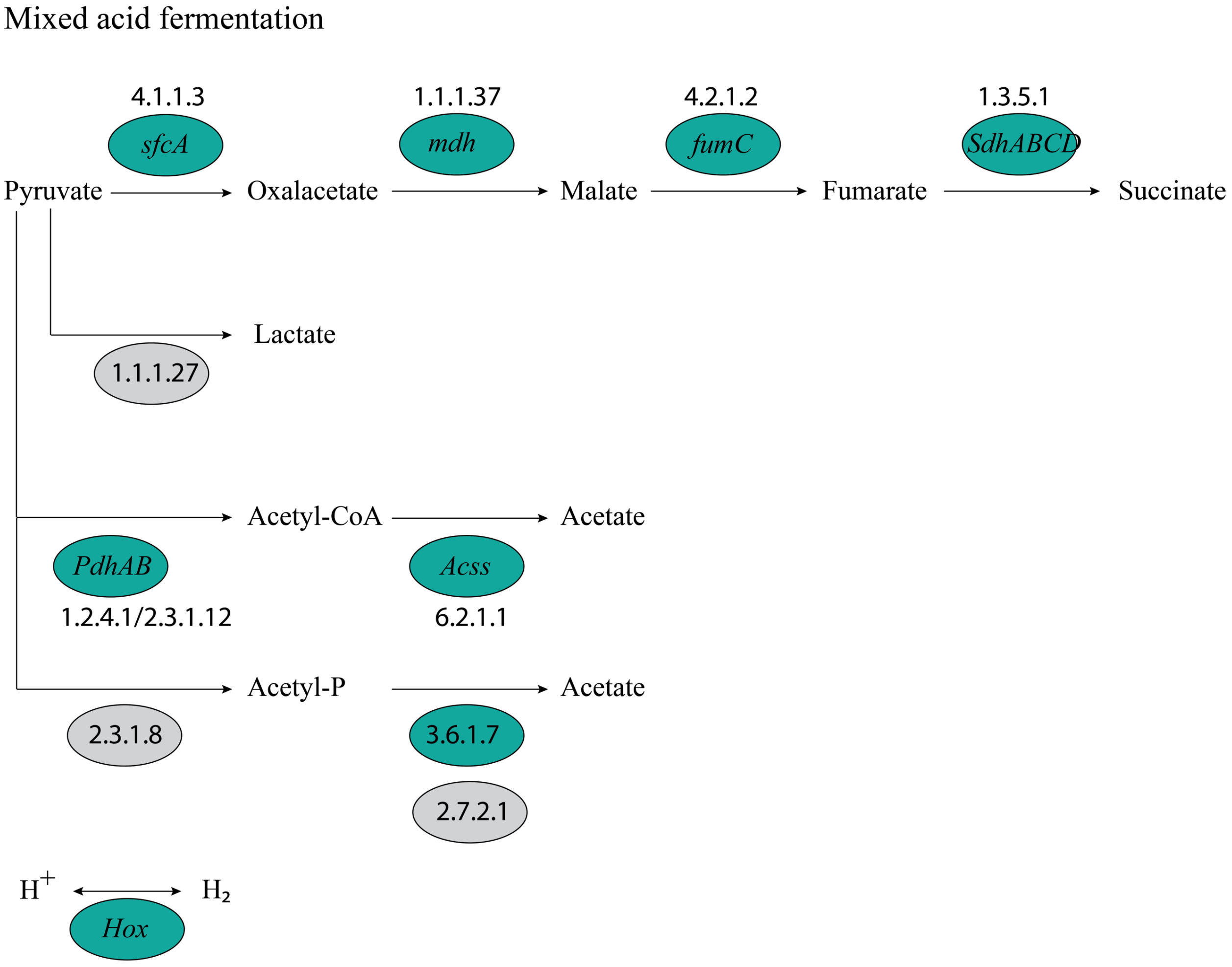

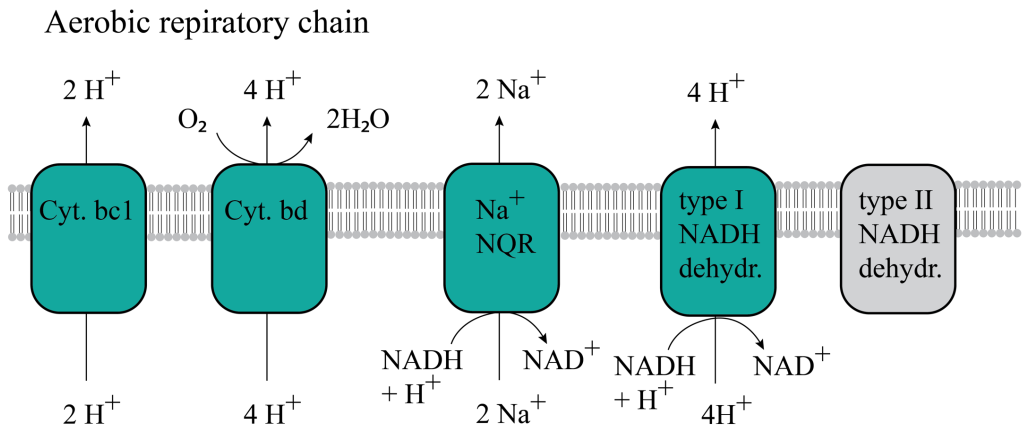

Fig. S5. Maximum likelihood phylogenetic tree based on 34 concatenated single-copy, protein-coding genes (following the method of Dombrowski et al., 2018) of the three highest average abundance MAGs (i.e. bin-63, bin-37, and bin-19) detected in the winter incubation experiment under anoxic conditions and amended with methane.

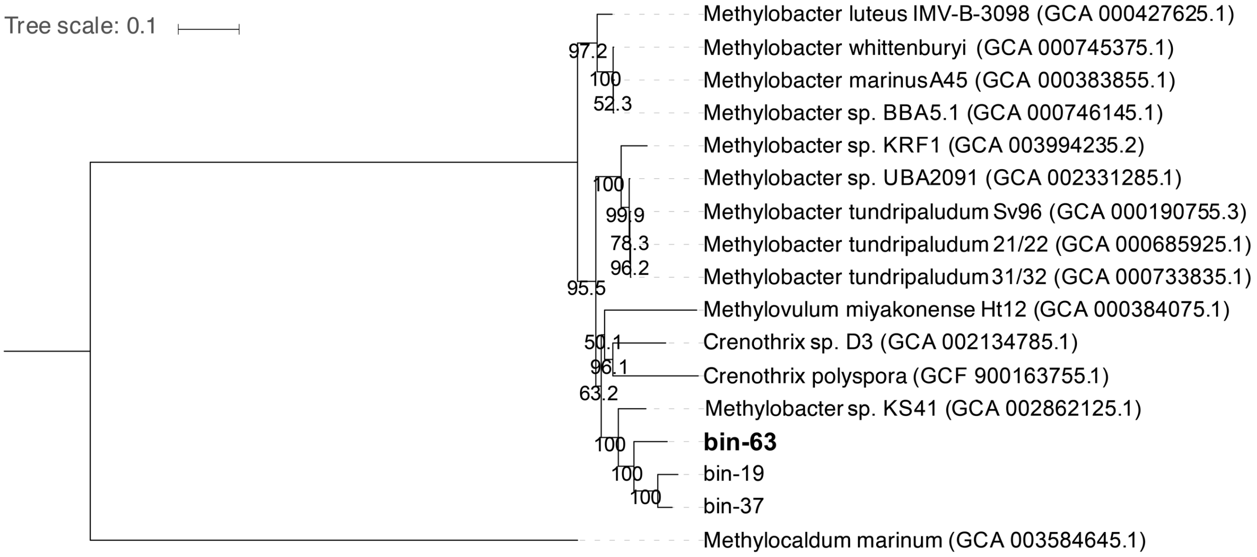

Fig. S6. GC coverage plots including the contigs of the 10 most abundant bins obtained in the sequenced sample with (A) the taxonomic classification of GTDB-Tk at the level of family, and (B) with the taxonomic classification of CheckM, indicating that the MAG bin-63, indicated in dark blue in panel B, was affiliated to the family Methylomonadaceae, indicated in teal in panel A.

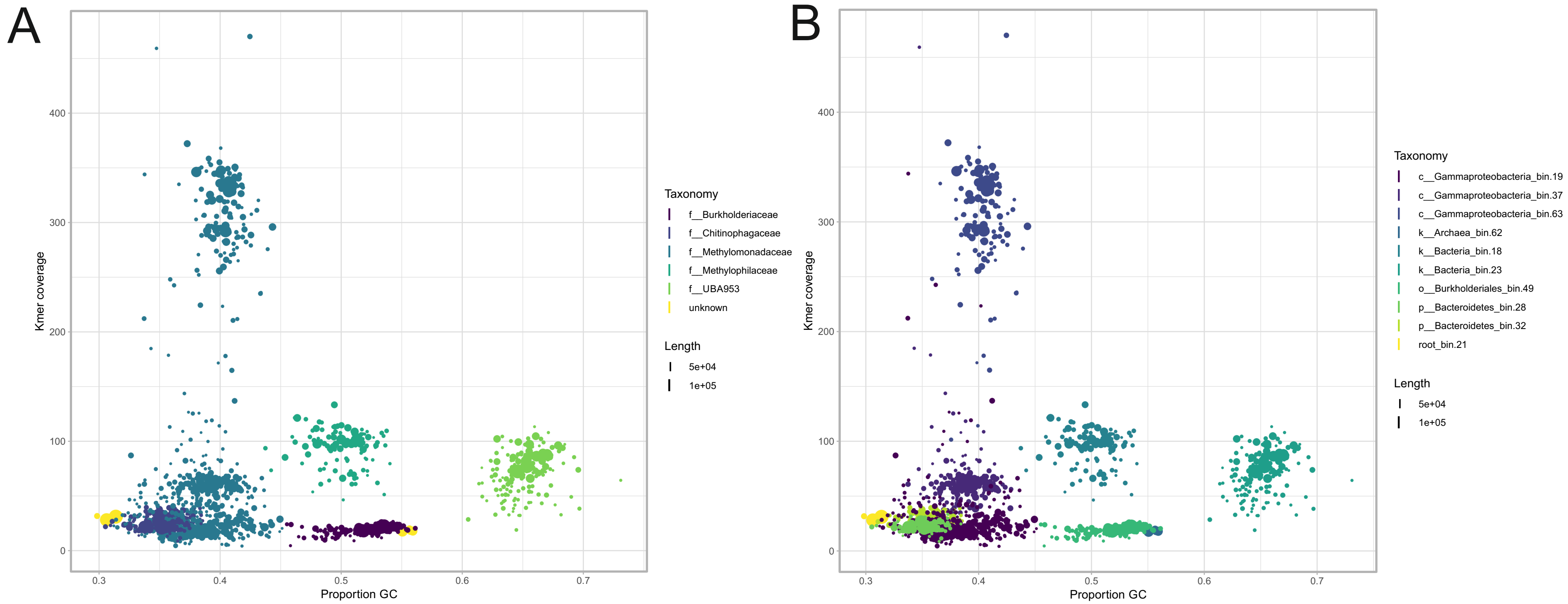

Table S1. Methane oxidation rates (MOR) and additional information regarding the rate measurements. The R^2 given is the R^2 of the linear regression analysis used to determine the methane oxidation rate. The oxidizing equivalents surplus/deficiency indicates the µM of methane that could have been oxidized by the electron acceptor, after the amount that was oxidized within the 24 h incubation experiment was deducted.

	Depth (m)	MOR (µM day^{-1})	t_0 $[CH_4]$	oxidized in 24 h (%)	R^2	$[NO_3^-]$ or $[SO_4^{2-}]$ (µM)	Potential CH_4 oxidation by $[NO_3^-]$ or $[SO_4^{2-}]$ (µM)[c]	Oxidizing equivalents surplus/ deficiency (µM)[c]
Summer, natural conditions	3	-	2.7	-	0.01			
	5	-	2.2	-	0			
	7	18	28	63	0.96			
	9	7.3	102	7	0.2			
	12	9.2	107	9	0.25			
	15	46	286	16	0.42[b]			
	17	36	398	9	0.21[b]			
Summer, nitrate addition	5	0.4	0.7	57	0.8	116	73	72
	7	30	9.6	100[a]	0.65	146	92	62
	9	58	113	51	0.98	124	78	20
	12	72	158	46	0.71	74	47	-26
	15	64	156	41	0.9	146	92	28
Summer, sulfate addition	5	0.5	0.7	70	0.08	2230	2230	2229
	7	13	9	100[b]	0.84	2159	2159	2150
	9	52	117	44	0.96	2272	2272	2155
	12	40	91	44	0.76	2267	2267	2176
	15	74	158	47	0.92	2194	2194	2037
Winter, methane addition	3	-	176	-	0.01			
	7	-	134	-	0.01			
	12	-16.4	168	-10	0.17			
	17	7.7[d]	151	5	0.06			
Winter, anoxic + methane addition	12	20.6	194	11	0.22			
	17	36.5	256	14	0.17			
Winter, natural	17	0.03[d]	0.84	3	0.08			

[a] the methane oxidation rate is based on the linear regression analysis, whilst the actual methane oxidation consumption in the incubation vials was lower due to methane limitation.

[b] a subset of the data points was used (t_6, t_{12} and t_{24})

[c] Using a 8:3 ratio of NO_3:CH_4 and a 1:1 ratio of SO_4:CH_4 (Segarra et al. 2013)

[d] Low R^2 of regression analysis

Table S2. Relative abundances (%) of known methanotrophs detected in the Lacamas Lake water column and incubation experiments (C denotes control) as determined by 16S rRNA gene amplicon sequencing. Any group with a percentage <0.1% is considered not significant and displayed as zero. Total reads obtained are indicated. Total 16S rRNA gene copies per liter were determined using quantitative PCR of the total prokaryotic community, with SD being the standard deviation of 3 experimental replicates. The taxonomic assignment of each of the mentioned methanotrophs is shown in Fig. 3.

	SUMMER										WINTER										
	Natural conditions (depth in m)							Incubations (12 m)			Natural conditions (depth in m)					Incubations (12 m)			Incubations (17 m)		
	3	5	7	9	12	15	17	C	NO_3^{2-}	SO_4^{2-}	3	5	12	15	17	C	CH4/ oxic	CH4/ anoxic	C	CH4/ oxic	CH4/ anoxic
CABC2E06	0	0.2	0.5	0.3	0.1	0.1	0.1	0.9	1	0.4	0	0	0	0	0	0	0.3	1	0	0.4	0.9
Crenotrix spp.	0	0	0.1	0.1	0	0	0	0.3	0.4	0.6	0	0.1	0	0	0.1	0	0.1	0.3	0	0.1	0.4
Methylobacter spp.	0	0.1	0.3	0.4	0.6	0.7	0.8	12	11	17	0.4	0.5	0.8	0.6	0.9	0.7	7	35	0.2	6.9	13
Methylomonas spp.	0	0.1	0.5	0.4	0.2	0.1	0.1	1.7	3.1	2.3	0	0	0	0.1	0	0	0	0	0	0	0
pLW-20	0	0.4	3.3	2.6	0.5	0.1	0	1.7	4	1.3	0	0	0	0.2	0	0	0.1	0.2	0	0	0.1
Unidentified *Methylococcaceae*	0	0.1	0.3	0.2	0.1	0	0	0.8	1.4	1.3	0	0	0	0	0	0	0	0	0	0.2	0.1
Reads per sample ($x10^6$)	1.7	1.4	1.9	1.8	0.9	1.5	1.7	1.5	1.8	1.5	8.8	1.7	1.4	2	1.6	1.7	1.6	1.3	1.4	1.3	1.5
Total 16S rRNA gene copies L^{-1} ($x10^8$)	4.1	5	4.6	5.4	5.5	6.8	3.1	10	3.2	3.3	2	3.4	2.8	3.1	3.6	7.1	4.8	11	1.6	2.2	2.4
SD	0.3	0.2	0.6	0.8	0.7	0.9	0.7	2.3	0.4	0.3	0.3	0.2	0.2	0.3	0.7	1.1	0.4	0.8	0.3	0.2	0.1

Table S3. *pmoA* relative abundance (%) in samples of the summer and winter water column and incubations (C denotes control). The tentative taxonomic assignment of the denovo sequences can be observed in Figure 5.

	SUMMER										WINTER										
	Natural conditions (depth in m)							Incubations (12 m)			Natural conditions (depth in m)					Incubations (12 m)			Incubations (17 m)		
	3	5	7	9	12	15	17	C	NO_3^{2-}	SO_4^{2-}	3	5	12	15	17	C	CH4/ oxic	CH4/ anoxic	C	CH4/ oxic	CH4/ anoxic
LL-pmoA-1	12	0.6	0.2	0.6	16	85	93	60	39	78	94	96	96	97	96	-	97	97	94	96	96
LL-pmoA-2	0.3	0	0	0	0.3	1.5	1.5	1.3	1.7	1.7	1.7	1.9	1.7	1.8	1.8	-	1.7	2	2.3	2.3	1.8
LL-pmoA-3	78	43	9.9	4.9	3.8	0.6	0.8	9.9	6.4	0.6	3.4	1.7	1.7	1.5	1.9	-	1.6	0.7	3.7	2.1	1.7
LL-pmoA-4	9.9	56	90	95	80	13	4.2	29	53	19	0.8	0.4	0.5	0.2	0.1	-	0.1	0.1	0.1	0	0.1

Table S4. Characteristics of the MAGs affiliated to methanotrophs of the Methylococcales which are discussed in the text. Classification was inferred by GTDB-Tk. An unassigned species (i.e., s__) indicates that the query genome is either i) placed outside a named genus or ii) the ANI to the closest intra-genus reference genome with an AF >=0.65 is not within the species-specific ANI circumscription radius. Classification was performed by placement of the genome in the reference tree and by using the relative evolutionary divergence (RED). Red value indicates the relative evolutionary divergence for a query genome. aa_percent: indicates the percentage of the multiple sequence alignment spanned by the genome (i.e. percentage of columns with an amino acid).

bin id	Completeness (%)	Contamination (%)	Strain heterogeneity (%)	contigs	size (bp)	Average abundance	aa_percent	red_value	classification
bin.63	96	0.74	0	136	2225455	419	97	0.95023	d__Bacteria;p__Proteobacteria; c__Gammaproteobacteria;o__Methylococcales; f__Methylomonadaceae;g__KS41
bin.37	92	5.3	14	282	2214630	77	87	0.951832	d__Bacteria;p__Proteobacteria; c__Gammaproteobacteria;o__Methylococcales; f__Methylomonadaceae;g__KS41
bin.19	88	0.46	50	453	4678414	30	74	0.951132	d__Bacteria;p__Proteobacteria; c__Gammaproteobacteria;o__Methylococcales; f__Methylomonadaceae;g__KS41

Table S5. Relative abundance (% of 16S rRNA gene reads) of *Sulfuritalea*, *Burkholderiales* and *Methylophilaceae* detected in Lacamas Lake by 16S rRNA gene amplicon sequencing (C denotes control).

	SUMMER										WINTER										
	Natural conditions (depth in m)							Incubations (12 m)			Natural conditions (depth in m)					Incubations (12 m)			Incubations (17 m)		
	3	5	7	9	12	15	17	C	NO_3^{2-}	SO_4^{2-}	3	5	12	15	17	C	CH4/ oxic	CH4/ anoxic	C	CH4/ oxic	CH4/ anoxic
Sulfuritalea (genus)	0.03	0.11	1.7	4.5	13	12	10	7.2	7.3	8.9	0.13	0.13	0.15	4.68	0.1	0.09	0.08	0.08	0.07	0.07	0.07
Burkholderiales (order)	4.5	7.1	14	15	17	11	8.6	12	8.7	11	28	23	35	22	28	19	17	8.5	19	15	10
Methylophilaceae (family)	0.85	1.4	2	1.3	1	0.64	0.69	3.7	5.2	3.7	0.72	0.7	1.2	1.1	1.5	0.94	2.6	6.8	1.4	3.1	3.1

Table S6. Experimental and sampling details. Samples for nutrient analysis were taken in duplicate (indicated with #), samples for methane oxidation rate linear regression analysis in quadriplicate (natural rate incubations indicated with +, amended incubations indicated with ^). For DNA analysis one filter per sampling moment and depth was taken (indicated with x).

	Nutrients						Microbial community		Incubations			
	Nitrate 0.2 μm filtered		Nitrite 0.2 μm filtered		Sulfate Zn-Acetate (0.4 mg l)		DNA samples 0.3 μm filtered		Methane oxidation rate 24-hour incubations		Methane oxidizing community 72-hour incubations, then 0.2 μm filtered	
Depth (m)	Summer	Winter	Summer	Winter	Summer	Winter	Summer	Winter	Summer	Winter	Summer	Winter
0												
1												
3	#	#	#	#	#	#		x	+	+		
5	#		#		#		x	x	+			
7	#	#	#	#	#	#	x		+	+		
9	#		#		#		x		+			
12	#	#	#	#	#	#	x	x	+ ^	+ ^	x ^	x ^
15	#	#	#	#	#	#	x	x	+			
17	#	#	#	#	#	#		x	+	+ ^		x ^
Inlet	#	#	#	#	#	#						

X. single sampling (for DNA purposes)
#. sampling in biological duplicate
+. sampling in biological quadruplicate

Table S7. List of 34 single-copy marker genes used for phylogenetic analysis of MAGs.

DNGNGWU00001
DNGNGWU00002
DNGNGWU00003
DNGNGWU00005
DNGNGWU00006
DNGNGWU00007
DNGNGWU00009
DNGNGWU00010
DNGNGWU00011
DNGNGWU00012
DNGNGWU00014
DNGNGWU00015
DNGNGWU00016
DNGNGWU00017
DNGNGWU00018
DNGNGWU00019
DNGNGWU00021
DNGNGWU00022
DNGNGWU00023
DNGNGWU00024
DNGNGWU00025
DNGNGWU00026
DNGNGWU00027
DNGNGWU00028
DNGNGWU00029
DNGNGWU00030
DNGNGWU00031
DNGNGWU00032
DNGNGWU00033
DNGNGWU00034
DNGNGWU00036
DNGNGWU00037
DNGNGWU00039
DNGNGWU00040

Table S8. Relative abundance of methanotroph OTUs as percentage of all reads in the sample (C denotes control). The taxonomic affiliation of the OTUs is shown in Figure 3.

	SUMMER										WINTER										
	Natural conditions (depth in m)							Incubations (12 m)			Natural conditions (depth in m)					Incubations (12 m)			Incubations (17 m)		
	3	5	7	9	12	15	17	C	NO_3^{2-}	SO_4^{2-}	3	5	12	15	17	C	CH4/ oxic	CH4/ anoxic	C	CH4/ oxic	CH4/ anoxic
LL-16S-01	0	0	0.33	0.29	0	0	0	0.11	0.04	0.01	0	0	0	0	0	0	0	0	0	0	0
LL-16S-02	0	0.01	0	0.06	0	0	0	0.02	0.06	0	0	0	0	0	0	0	0	0	0	0	0.01
LL-16S-03	0	0	0.03	0.06	0	0	0	0	0.01	0.04	0	0	0	0	0	0	0	0	0	0	0
LL-16S-04	0	0	0.51	0.08	0	0	0	0	0	0.02	0	0	0	0	0	0	0	0	0	0	0
LL-16S-05	0	0.02	0.02	0.08	0.02	0	0	0.1	1	0.04	0	0	0	0	0	0	0	0	0	0	0
LL-16S-06	0	0.01	0.08	0.25	0	0	0	0.02	0.01	0.08	0	0	0	0	0	0	0	0	0	0	0.01
LL-16S-07	0	0	0	0.07	0	0	0	0.01	0.01	0.01	0	0	0	0	0	0	0	0	0.01	0.86	0.05
LL-16S-08	0	0	0	0	0	0	0	0.01	0.02	0.01	0	0	0	0	0	0	0	0	0	0	0
LL-16S-09	0	0	0	0	0	0	0	0.01	0.01	0.01	0	0	0	0	0	0	0	0	0	0	0.02
LL-16S-10	0	0	0	0	0	0	0	0	0	0	0	0	0	0	0	0	0	0	0	0.01	0.01
LL-16S-11	0	0.01	0.01	0.02	0	0	0	0.14	0.2	0.01	0	0	0	0	0	0	0	0	0	0	0
LL-16S-12	0	0.01	0	0	0	0	0	0.01	0.02	0	0	0	0	0	0	0	0	0	0	0.01	0.01
LL-16S-13	0	0	0	0	0	0	0.01	0.04	0.12	0.03	0	0	0	0	0	0	0.01	0.03	0	0	0
LL-16S-14	0	0	0	0	0	0	0	0	0	0	0	0	0	0	0	0	0	0	0	1.3	5
LL-16S-15	0	0	0	0	0	0	0	0	0	0	0	0	0	0	0	0	0	0	0	0	0
LL-16S-16	0.01	0	0.01	0.02	0.02	0.09	0.01	1.8	1.1	4.7	0.03	0.01	0.08	0.04	0.02	0.09	0.03	17	0	0	0
LL-16S-17	0	0	0	0	0.01	0.01	0.07	0.01	0.51	0.01	0	0.04	0	0.01	0.01	0	1.8	0.04	0	0	0
LL-16S-18	0	0	0	0	0	0	0	0.05	0.25	0.03	0	0	0	0	0	0	0	0	0	0	0
LL-16S-19	0	0	0	0	0	0	0	0	1.1	0.01	0	0	0	0	0	0	0.06	0.02	0	0	0
LL-16S-20	0	0	0	0	0	0	0	0.12	0.29	0.04	0	0	0.01	0	0	0	0	0.03	0	0	0
LL-16S-21	0	0	0	0	0	0.03	0	1.8	0.88	0.08	0.01	0.01	0	0	0	0	0	0.81	0	0	0
LL-16S-22	0	0	0	0	0	0	0	0.12	0.29	0.04	0	0	0.01	0	0	0	0	0.03	0	0	0
LL-16S-23	0	0	0	0	0.02	0	0.03	0.12	0.01	0.04	0	0	0.03	0.03	0.13	0.01	0.65	0.47	0	0.1	0.06
LL-16S-24	0	0	0	0	0	0	0.01	0.24	0.33	0.13	0	0	0	0	0	0	0.01	0.04	0.01	0.08	0.01
LL-16S-25	0	0	0	0	0	0	0	0	0	0	0	0	0	0	0	0	0	0	0.02	0	0.01
